# Novel CD44-Targeted Albumin Nanoparticles: An Innovative Approach to Improve Breast Cancer Treatment

**DOI:** 10.3390/ijms251910560

**Published:** 2024-09-30

**Authors:** Giuseppe Cirillo, Anna Rita Cappello, Manuela Curcio, Marco Fiorillo, Luca Frattaruolo, Paola Avena, Ludovica Scorzafave, Vincenza Dolce, Fiore Pasquale Nicoletta, Francesca Iemma

**Affiliations:** Department of Pharmacy Health and Nutritional Science, University of Calabria, 87036 Rende (CS), Italy; giuseppe.cirillo@unical.it (G.C.); annarita.cappello@unical.it (A.R.C.); luca.frattaruolo@unical.it (L.F.); paola.avena@unical.it (P.A.); ludovicascorzafave@gmail.com (L.S.); fiore.nicoletta@unical.it (F.P.N.); francesca.iemma@unical.it (F.I.)

**Keywords:** breast cancer, ionic nanoparticles, hyaluronic acid, cationized albumin, vectorization

## Abstract

This study introduces novel CD44-targeted and redox-responsive nanoparticles (FNPs), proposed as doxorubicin (DOX) delivery devices for breast cancer. A cationized and redox-responsive Human Serum Albumin derivative was synthesized by conjugating Human Serum Albumin with cystamine moieties and then ionically complexing it with HA. The suitability of FNPs for cancer therapy was assessed through physicochemical measurements of size distribution (mean diameter of 240 nm), shape, and zeta potential (15.4 mV). Nanoparticles possessed high DOX loading efficiency (90%) and were able to trigger the drug release under redox conditions of the tumor environment (55% release after 2 h incubation). The use of the carrier increased the cytotoxic effect of DOX by targeting the CD44 protein. It was shown that, upon loading, the cytotoxic effect of DOX was enhanced in relation to CD44 protein expression in both 2D and 3D models. DOX@FNPs significantly decrease cellular metabolism by reducing both oxygen consumption and extracellular acidification rates. Moreover, they decrease the expression of proteins involved in the oxidative phosphorylation pathway, consequently reducing cellular viability and motility, as well as breast cancer stem cells and spheroid formation, compared to free DOX. This new formulation could become pioneering in reducing chemoresistance phenomena and increasing the specificity of DOX in breast cancer patients.

## 1. Introduction

Cancer, a major problem in the 21st century, is among the three leading causes of death in patients aged between 30 and 69 years worldwide [1]. It impacts society from societal, public health, and economic points of view, varying in degree across cancer types, geography, and gender [2]. According to the latest GLOBOCAN estimates produced by the International Agency for Research on Cancer, female breast cancer is the second leading cause of global cancer incidence in 2022, comprising 11.6% of all cancer cases and 6.9% of all cancer deaths. Being the most commonly diagnosed cancer, it is the leading cause of cancer deaths globally [3]. The assessment of the global burden of disease and temporal trends of breast cancer proved that between 1990 and 2019, the incident cases increased from 876,990 to 2,002,350, and the estimated annual percentage change for incidence increased each year by 0.33% [4]. These dramatic data claim for the development of specific and innovative therapies to reduce the burden of morbidity and mortality associated with breast cancer [5].

Nanotechnologies offer promising tools in modernizing cancer diagnosis and treatment [6,7], particularly in optimizing the chemotherapeutic protocols via the vectorization of the selected therapeutic to the target site [8]. To date, a plethora of nanoparticle systems loaded with cytotoxic drugs have been developed as a promising alternative to conventional chemotherapy, by virtue of both the effective accumulation in the tumor site closely related to the nanometric size (Enhanced Permeation and Retention—EPR effect) and the ability to protect cargo from harsh environments by controlling the release over time [9,10]. These delivery systems, defined as passively targeted formulations, often suffer from some limitations related to off-target drug accumulation, due to the lack of uptake control and to the high heterogeneity of the tumor vessels’ permeability, with the possibility to cope with the onset of multidrug resistance phenomena [11,12]. The breakthrough was made by developing a new generation of actively targeted nanosized platforms, also defined as smart materials, which act by recognizing the cancer site by interaction with peculiar cellular components (i.e., membrane receptors) [13,14] or by responding to specific signals coming from the tumor microenvironment (e.g., variation in pH, temperature, enzyme concentration, redox potential) [15,16]. Ultimately, the development of smart nanoparticles able to overcome biological barriers (systemic, microenvironmental, and cellular) and selectively reach the target site opens new perspectives for the treatment of cancer through the implementation of so-called precision medicine, with considerable advantages compared to conventional therapies [17,18].

Among the different membrane receptors, CD44 represents an attractive target for cancer treatment due to its higher expression in many cancer cells (i.e., breast, lungs, ovary, brain, and hepatic carcinoma) vs. normal cells [19], where it plays a key role in cell proliferation, differentiation, and cell migration [20]. CD44 has strong affinity to O-glycans, N-glycans, and glycosaminoglycans, such as chondroitin sulfate, heparan sulfate, and hyaluronic acid (HA) [21]. Different studies proved that the derivatization of nanoparticle systems with the CD44 native ligand (HA) is a valuable approach to selectively target cancer cells, thus enhancing the uptake of a loaded therapeutic agent [22,23,24].

On the other hand, the literature’s data clearly show that the highly reducing properties of the tumor environment, related to the high intracellular concentration of glutathione (GSH), facilitate the release of the payload into the cytosol of cancer cells by inducing chemical changes in the redox-responsive entities within the nanocarrier. As a consequence, the nanoparticle’s structure is destabilized and the cytotoxic drug can directly and more effectively interact with the target [25,26].

Over the last decades, smart nanoparticle platforms with different architectures and compositions have been fabricated from a large number of biomaterials, and, among them, functional composites of natural polymers have attracted much interest due to their high biocompatibility and chemical versatility [27,28]. In this regard, Human Serum Albumin (HSA) has attracted great interest due to its high loading capability [29,30], and several targeted redox-responsive nanocarriers have been prepared by tailored derivatization of HSA with disulfide functionalities [31,32].

In this work, with the aim to combine the CD44-targeting ability of HA and the redox-responsivity of cystamine (cys), we develop a nanoparticle system (FNPs) via ionic complexation at physiological pH of cysHSA derivative and HA for doxorubicin (DOX) vectorization in breast cancer cell lines. This approach is a key innovation over the strategies explored in the literature reporting on the use of either unmodified HSA ionically complexed with HA at non-physiological pH values [33] or cationized HSA formulated in nanoparticles via emulsion techniques and subsequently decorated with HA moieties [34]. To the best of our knowledge, this is the first example of a cationized derivative of HSA acting, at the same time, as a branch for complexing HA and as a redox-responsive element.

The suitability of FPNPs for application in cancer therapy was evaluated by physicochemical determination of shape, size distribution, and zeta potential measurements. Then, we investigated the effects of DOX-loaded nanoparticles on breast cancer cell lines with different CD44 receptor expression. CD44, a pivotal adhesion molecule, is involved in diverse biological processes like cell migration, proliferation, tumor development, and metastasis [35]. It acts as a surface marker for cancer stem cells, which are capable of initiating tumors in some types of cancers. It has been demonstrated that CD44 plays a role in regulating the glycolytic pathway in colorectal cancer and lung carcinoma cell lines. Moreover, the regulation of glucose metabolism by CD44 contributes to antioxidant status and drug resistance in cancer cells [36]. This indicates that CD44 may potentially modulate the behavior of triple-negative breast cancer cells (TNBCs), known for their high glycolytic activity.

## 2. Results and Discussion

### 2.1. Synthesis and Characterization of Cationic cysHSA

**Figure 1:**
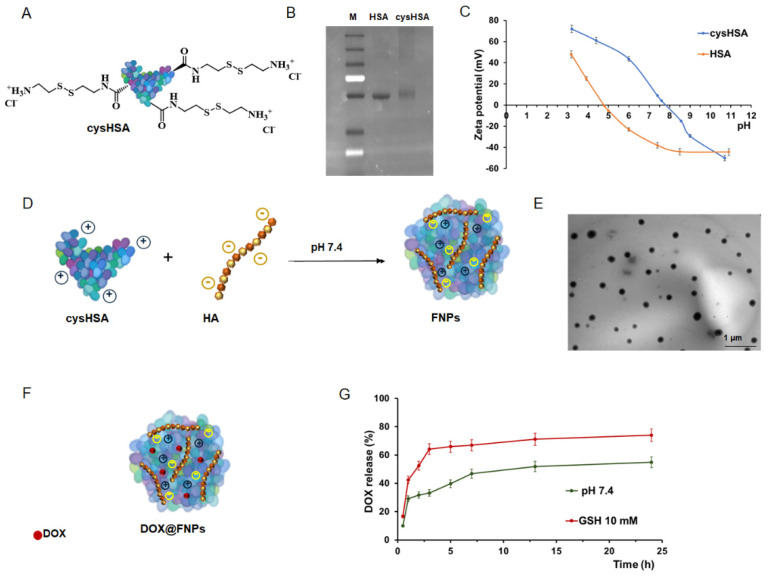
Preparation and characterization of Human Serum Albumin derivative (cysHSA), redox-responsive nanoparticle (FNPs), and doxorubicin-loaded FNPs (DOX@FNPs). (**A**) Schematic representation of cysHSA conjugate; (**B**) SDS page of HSA and cysHSA under not-reducing conditions; (**C**) determination of PI for Human Serum Albumin (HSA, orange) and cysHSA (blue) by zeta potential measurements; (**D**) formation of FNPs by ionic complexation of cysHSA (positive charge) and Hyaluronic acid (HA, negative charge)) at physiological pH; (**E**) representative TEM image of negatively stained FNPs; (**F**) schematic representation of DOX@FNPs; (**G**) DOX release profiles from DOX@FNPs at physiological (green) vs. redox (red) conditions.

Cationized HSA (cysHSA, Figure 1A) was synthesized by exploiting the coupling reaction between the aspartate and glutamate side groups of native HSA and the amine groups of proper derivatizing specimen (cysHCl) using EDC as a condensing agent [37].

To maximize the substitution degree and the product yield, the reaction was carried out at the Isoelectric Point (IP) of the protein (pH 4.8), and different cysHCl-to-HSA (in terms of mol of Glutamic acid and Aspartic acid) ratios (%) were explored (from 1 to 5%), with a value of 2.7% found to be the optimal condition. A further increase in cysHCl was found to be not effective in enhancing the cystamine content, probably as a consequence of steric hindrance phenomena.

After purification by dialysis, successful derivatization was confirmed by SDS-PAGE under not-reducing conditions to avoid any interference with the linked cys moieties [38]. As displayed in Figure 1B, as expected for not-reducing conditions, a slight increase in the molecular weight was recorded for cysHSA vs. native protein migrating at 55 kDA, due to the addition of cys residues to the protein backbone.

Moreover, TNBS allowed a 31% increase in the amino group content in cysHSA compared to native HSA to be calculated. Finally, ζ measurements, performed at pH values ranging from 3 to 11, displayed a shift in IP value from 4.8 ± 0.5 to 7.8 ± 0.8 for native HSA and cysHSA, respectively, suggesting that the positive charge of cysHSA is maintained over a broad pH range including the physiological conditions (Figure 1C). As expected, when the pH reaches the IP value of cysHSA, aggregation phenomena are observed (the solution turns slightly opalescent) as a consequence of the neutralization of the positive surface charges of cysHSA.

### 2.2. Synthesis and Characterization of FNPs

In order to fabricate smart ionically crosslinked nanoparticles, HSA endowed with redox functionalities (cysHSA) was combined with an anionic polysaccharide such as HA at physiological pH by directly adding HA to cysHSA solution until an opalescent colloidal suspension was formed (Figure 1D). By fixing the cysHSA-to-HA ratio to 6:1 (*w*/*w*), monodispersed spherical particles with an average hydrodynamic diameter of 240 ± 35 nm (P.D.I. of 0.2) were observed, as per DLS and TEM analyses, with no interference from the contrast agent (Figure 1E). These dimensional parameters were well fit with the requirements of an ideal drug delivery system able to penetrate tumor tissues via the EPR effect [39]. A higher protein-to-polysaccharide ratio did not allow the formation of stable nanoparticles; otherwise, larger nanostructures (~400 nm), not suitable as drug delivery vehicles for anticancer therapy, were observed when an increased amount of HA was used. Moreover, FNPs possessed a surface charge of 15.4 mV (as per ζ-potential measurements), suggesting good colloidal stability by virtue of the negligible particle aggregation phenomena [40], as confirmed by stability tests over time (7 days at 25 °C).

### 2.3. Drug Delivery Studies

A nanocarrier designed for drug vectorization into cancer cells should be able to selectively recognize the target and release its cargo in response to specific environmental signals, saving healthy cells from the cytotoxic effects of chemotherapy [41,42]. DOX, one of the most effective antineoplastic drugs used for breast cancer treatment [43], was loaded into FNPs (Figure 1F) during the self-assembly process, exploiting the strong affinity between DOX and HSA [44,45]. The drug (22 mg per g of cysHSA) was incubated in cysHSA solution before the addition of HA, and a high loading efficiency (90%) was calculated. Moreover, the unchanged morphological and dimensional parameters of DOX@FNPs compared to unloaded systems demonstrated that the complexation process was not affected by the drug. The amount of DOX released from DOX@FNPs was determined in phosphate buffer at pH 7.4 and GSH 10 mM, simulating the extracellular and intracellular space conditions, respectively [46,47]. Data depicted in Figure 1G allow for hypothesizing the effective destabilizing effect of GSH on the nanoparticle structure: at pH 7.4, indeed, the DOX cumulative release did not exceed 55% after 24 h, whereas the presence of GSH sped up the release, with the same percentage being recorded just after 2 h. This result can be ascribable to the reducing activity of GSH on the disulfide bonds, which causes a weakening of the whole nanoparticle matrix and an easier drug diffusion in the release medium.

### 2.4. Safety and Toxicity of DOX@FNPs in Breast Cancer Cell Lines

**Figure 2:**
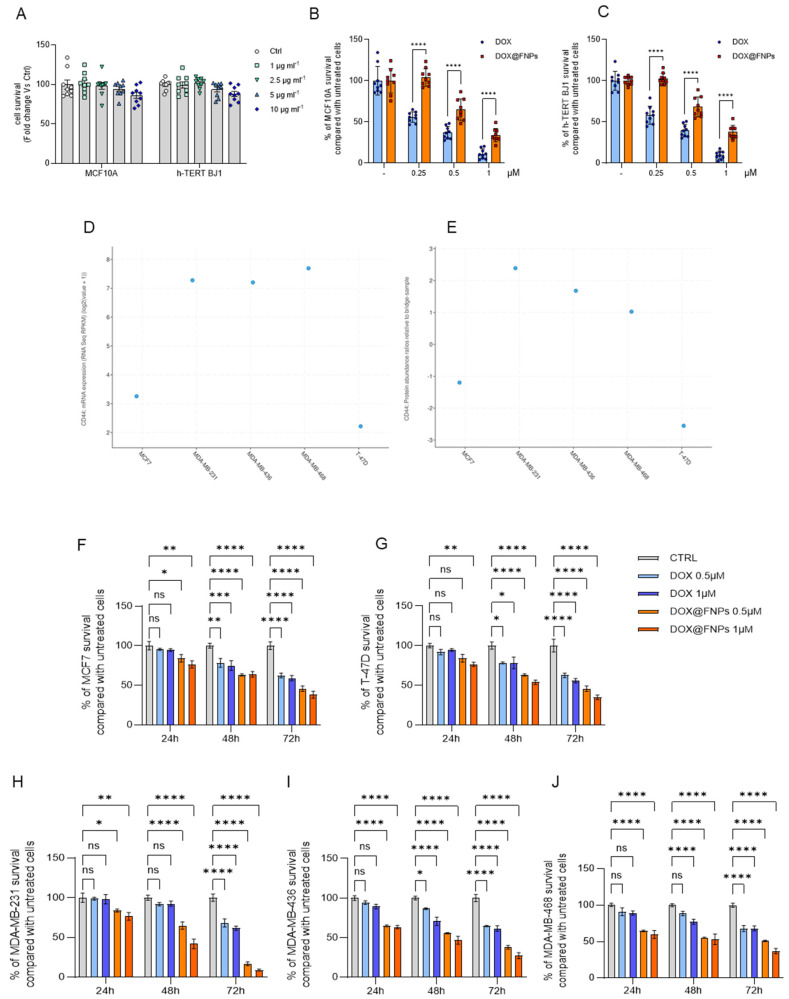
The cell viability evaluation of FNPs and DOX@FNPs. (**A**) The cytotoxic effect of FNPs vehicle alone on non-cancerous cells MCF10A and h-TERT BJ1. Note that no significant effects were detected after treatment with high concentrations of FNPs. The effect of DOX and DOX@FNPs on non-cancerous cells (**B**) MCF10A and (**C**) h-TERT BJ1; One-way ANOVA, **** *p*-value <0.0001. (**D**,**E**) Panel (**C**) shows the mRNA levels of the glycoprotein CD44, while Panel (**D**) shows the protein expression of CD44. The data were obtained through bioinformatic analysis on www.cbioportal.org, accessed on 1 June 2024. (**F**–**J**) MTT viability assay performed on 2D cultures. It is possible to note the significance of the effect of DOX@FNPs, especially after 72 h of treatment. Two-way ANOVA. ns: not significant; * *p*-value < 0.05; ** *p*-value < 0.01; *** *p*-value < 0.001; **** *p*-value < 0.0001.

First, to validate the safety of the formulation, the toxicity of FNPs was tested on two non-tumor cell lines, MCF10A and h-TERT-BJ1. FNPs did not show any toxic effect even at high concentrations (10 μg mL^−1^) in both the tested cell lines (Figure 2A). Further, the toxicity of free DOX and DOX@FNPs was evaluated on the same cells at the equivalent drug concentrations (from 0.25 to 1.0 μM), and a lower toxicity for loaded vs. free DOX was observed, suggesting a better safety profile (Figure 2B,C).

Using bioinformatics data, CD44 gene transcription and protein expression were measured in multiple cancer types. Moreover, CD44 expression was quantified in normal, tumor, and metastatic tissue (www.cbioportal.org, see Section 3, Supplementary Figure S1A–C). The mRNA (Figure 2D) and protein (Figure 2E) expression levels of CD44 were also evaluated in the tested breast cancer cell lines, with TNBC cell lines expressing higher levels of CD44 transcript and protein than ER+ cell lines.

The results of cytotoxicity assays are consistent with the involvement of CD44 in the DOX@FNPs’ toxicity on cancer cells: DOX@FNPs had a more pronounced effect on TNBC cells expressing higher levels of the CD44 protein, compared to the free DOX (Figure 2F–J). After 72 h incubation, indeed, free DOX (1 μM) was found to reduce the MCF-7 and T-47D viability by around 60%, with DOX@FNPs enhancing this effect up to 45%. On the other hand, the enhancing effect of nanoparticles was more evident when TNBC cell lines were used as an in vitro model, with the viability moving from almost 65% (MDA-MB-231, MDA-MB-436, MDA-MB-468), after adding DOX, to 8% (MDA-MB-231), 27% (MDA-MB-436), and 37% (MDA-MB-468), after DOX@FNPs treatment. The results suggest a higher specificity of DOX@FNPs towards TNBC cell lines.

The enhancement of DOX cytotoxic activity can be ascribed to the selective interaction of HA moieties on the FNPs’ surface with the CD44 receptors on the cell membrane, the uptake of the nanoparticle system by cancer cells, and the subsequent increase in the DOX release rate in the redox conditions in the intracellular compartment.

### 2.5. DOX@FNPs Affected Cellular Metabolism by Targeting CD44

**Figure 3:**
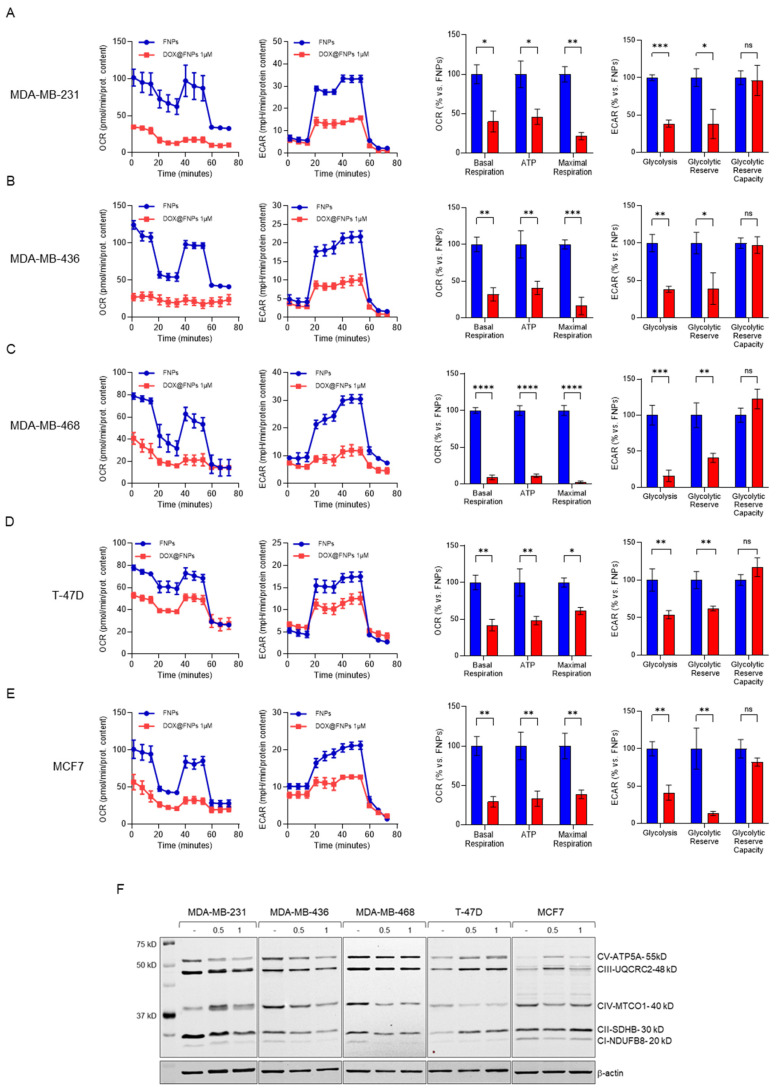
The metabolic profiles of breast cancer cell lines after treatment with DOX@FNPs. Panels (**A**–**E**) show the metabolic profiles of various cell lines obtained with the Seahorse Analyzer XFe96. OCR and ECAR were monitored in the presence of DOX@FNPs for 72 h. The bar graphs represent the levels of glycolysis, glycolytic reserve, and glycolytic capacity obtained from the ECAR analysis. Additionally, basal respiration levels, proton leak, ATP production, and maximal respiration were evaluated. One-way ANOVA, ns: not significant; * *p*-value < 0.05; ** *p*-value < 0.01; *** *p*-value < 0.001; **** *p*-value < 0.0001. (**F**) Western Blotting analysis of the expression levels of subunits involved in mitochondrial respiration and oxidative phosphorylation after 72 h treatment with DOX@FNPs.

TNBC cells are more glycolytic than ER+ cell lines. Thus, we evaluated the activity of DOX@FNPs after 72 h of treatment on a panel of tumor cell lines in comparison with DOX using a Seahorse Analyzer XF96 (Supplementary Figure S1D). The results revealed that, after treatment with DOX@FNPs, ECAR was more significantly inhibited in TNBC cell lines (MDA-MB-231, MDA-MB-436, MDA-MB-468) compared to ER+ tumor cell lines (Figure 3A–E). The levels of glycolysis, glycolytic reserve, and glycolytic reserve capacity were in fact strongly reduced after treatment with DOX@FNPs. By markedly reducing ECAR levels in cells dependent on glucose metabolism, the inhibitory effect of DOX@FNPs also affected OCR, severely reducing the basal and maximal respiration, as well as the ATP production. The significant decrease in OCR levels prompted us to investigate whether the mitochondrial metabolism and, in particular, the oxidative phosphorylation were affected. By Western Blot analysis, it was proved that DOX@FNPs impaired multiple complexes involved in the electron transport chain (Figure 3F), with a more pronounced effect in TNBC cell lines expressing high levels of CD44 protein compared to ER+ cell lines, corroborating the marked reduction in ECAR and OCR levels induced by DOX@FNPs.

### 2.6. DOX@FNPs Inhibited 2D Migration and 3D Tumor Formation

**Figure 4:**
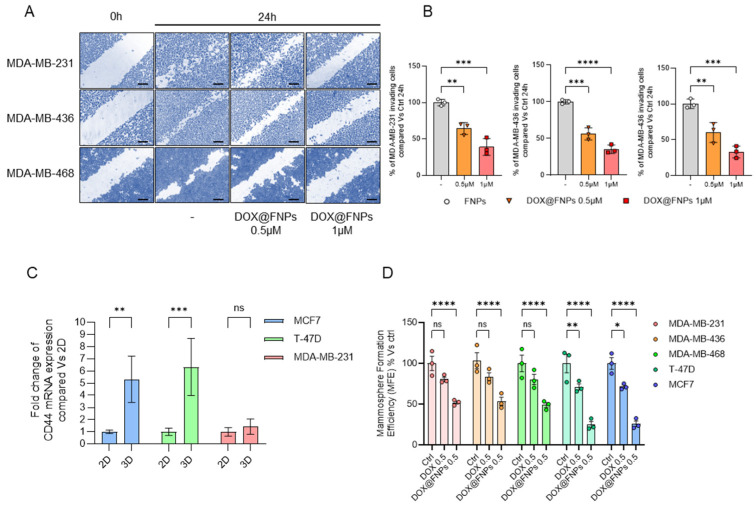
Invasiveness and stemness evaluation of breast cancer cell lines treated with DOX@FNPs. (**A**,**B**) Scratch assay on TNBC cells treated with DOX@FNPs for 24 h. After treatment, migratory capacity of the cells is significantly reduced. Scale bar 100 μm. One-way ANOVA, ** *p*-value < 0.01; *** *p*-value < 0.001; **** *p*-value < 0.0001. (**C**) Expression of CD44 transcript in 2D and 3D models of MCF7, T-47D, and MDA-MB-231 cells. Two-way ANOVA ** *p*-value < 0.01; *** *p*-value < 0.001. (**D**) Mammosphere formation efficiency of breast cancer cell lines treated with DOX 0.5 µM and DOX@FNPs 0.5 µM for 5 days. Two-way ANOVA, ns: not significant; * *p* value < 0.05; ** *p*-value < 0.01; **** *p*-value < 0.0001.

TNBC cells are known for their high aggressivity and rapid migration capability, contributing to the phenomenon of spreading, which initiates metastasis. Thus, a wound-healing assay was carried out to investigate the ability of DOX@FNPs to reduce the TNBC invasiveness, demonstrating that DOX@FNPs reduce the migration of TNBC cells in a concentration-dependent manner after 24 h of treatment. As illustrated in Figure 4A, the wound-healing capacity of all cell lines was significantly reduced after treatment with DOX@FNPs compared to the control, with a percentage of closure inhibition, measured 24 h after nanoparticles treatment (Figure 4B), of about 40% and 55% for 0.5 and 1 μg mL^−1^ DOX equivalent concentrations, respectively. Overall, these results suggested that DOX@FNPs might have a potential to inhibit invasiveness and thus the metastatic potential of TNBC.

The ability of tumor cells to form three-dimensional structures under no-attachment conditions, mimicking tumor or metastatic structures, enhances their malignancy. Through Western Blot analysis, we evaluated the expression levels of the CD44 protein, a stemness marker, in both 2D and 3D cancer cells used in this study. The results showed that 3D ER+ cell lines expressed higher levels of CD44 compared to 2D, while the levels of CD44 in 3D TNBC do not increase drastically compared to 2D TNBC (Figure 4C). Thus, we investigated the effect of DOX@FNPs on the formation of cancer stem cells (CSCs), particularly focusing on the inhibition of mammosphere formation efficiency (MFE). First, we compared the effect of DOX and DOX@FNPs, showing that DOX@FNPs had a strongly inhibitory effect on MFE compared to DOX. Secondly, we demonstrated that in 3D ER+cell lines, DOX@FNPs significantly reduced MFE in a concentration-dependent manner compared to TNBC cell lines (Figure 4D).

**Figure 5:**
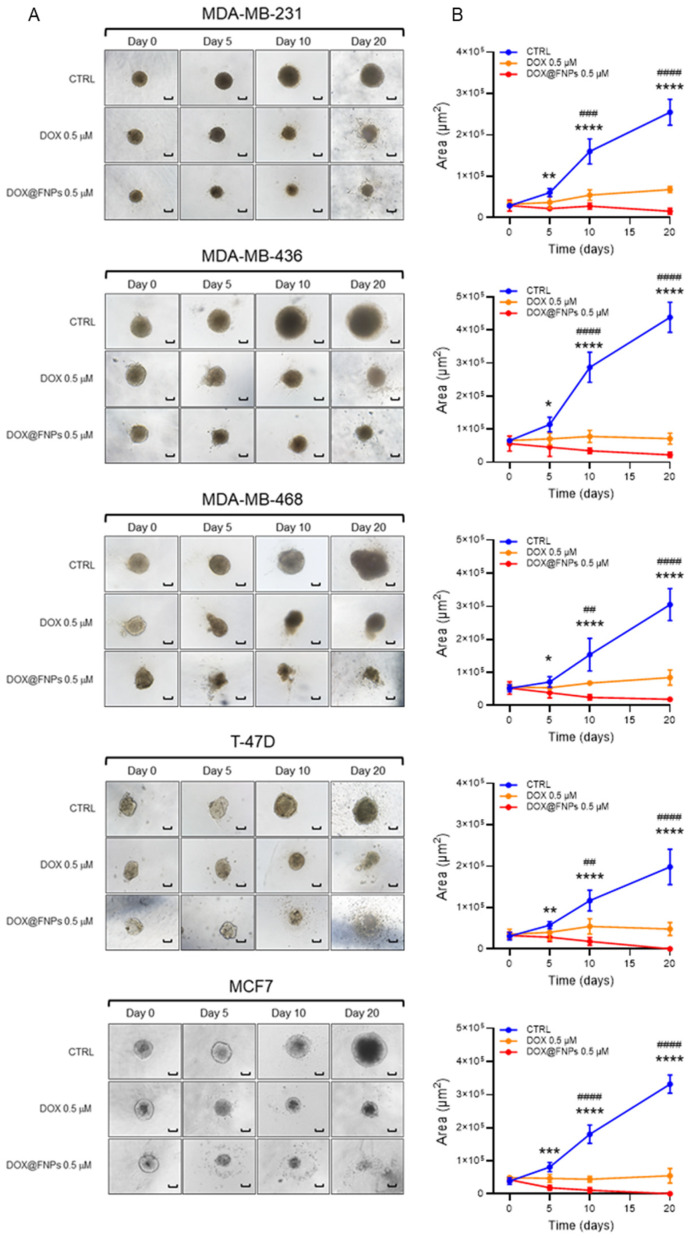
Three-dimensional spheroid analysis after treatment with DOX@FNPs. (**A**) Spheroid formation of all breast cancer cell lines treated with DOX and DOX@FNPs. Scale bar 50 μm. (**B**) The significant effect at 20 days demonstrated that DOX@FNPs 0.5 µM, compared to DOX, can significantly reduce spheroid formation in MDA-MB-231, MDA-MB-436, and MDA-MB-468 cell lines. The spheroids generated from T-47D and MCF7 cell lines were completely disintegrated after 20 days of treatment with DOX@FNPs 0.5 µM. DOX vs. CTRL: One-way ANOVA, ## *p*-value < 0.01; ### *p*-value < 0.001; #### *p*-value < 0.0001. DOX@FNPs vs. CTRL: One-way ANOVA, * *p*-value < 0.05; ** *p*-value < 0.01; *** *p*-value < 0.001; **** *p*-value < 0.0001.

Additionally, we tested the ability of DOX@FNPs to reduce the capacity of tumor cells to form spheroids (Figure 5A).

After generating spheroids from all the cell lines, we treated them with DOX and DOX@FNPs, extending the treatment for 20 days. The results showed that DOX@FNPs significantly reduced the volume of spheroids after only 5 days of treatment (Figure 5B). Furthermore, the treatment with DOX@FNPs completely disintegrated the spheroids formed from ER+ cell lines expressing the highest levels of CD44.

This result suggested that DOX@FNPs is more effective in inhibiting the formation of mammospheres and spheroids in ER+ cell lines, highlighting their potential to target and reduce the stemness and malignancy of certain breast cancer cells, thus emphasizing their utility in treating cancers characterized by high levels of CD44 expression.

For assessing the possible bench-to-clinic translation of the proposed formulation, further experiments will involve a more extensive characterization of the effects at the cellular level (e.g., biomechanical alterations [48,49]), as well as the determination of both pharmacokinetic profiles [50] and anticancer efficiency in suitable in vivo models [51].

## 3. Materials and Methods

### 3.1. Synthesis and Characterization of cysHSA Conjugate

A total of 2.73 g (0.01 mmol) cystamine dihydrochloride (cysHCl) in buffer solution (6 mL, 10^−3^ M, pH 4.78) was added to an aqueous solution (5 mL) containing HSA (0.25 g) and 1-ethyl-3-(3-dimethylaminopropyl) carbodiimide (EDC, 0.05 g, 0.032 mmol). The mixture was reacted for 4 h at room temperature under continuous magnetic stirring. The obtained cysHSA conjugate was purified by dialysis (SpectraPor, MWCO 12–14 kDa, VWR, Milan, Italy) against water at 20 °C for 72 h and recovered by freeze-drying (93% yield). The (2,4,6)-trinitrobenzenesulfonic acid (TNBS) assay was used to estimate the derivatization degree, in terms of increased amino group content, by the determination of free amino groups in HSA and cysHSA, respectively. Briefly, 0.5 mL 2,4,6-trinitrobenzenesulfonic acid solution (0.01% *w*/*v*) was added to 1.0 mg NaHCO_3_ (0.1 mol L^−1^, pH 8.5) containing has or cysHSA (2.0 mg mL^−1^) and, after mixing in the dark, placed in a water bath at 37 °C for 4 h. Then, sodium dodecyl sulfate (SDS, 50 μL, 0.35 mol L^−1^) and HCl (25 μL, 1.0 mol L^−1^) were added to stop the reaction. The absorbance was measured at 335 nm on an Evolution 201 spectrophotometer (ThermoFisher Scientific, Hillsboro, OR, USA). *CDD* (%) was calculated according to the following Equation (1):

(1) $$CDD\left(\%\right)=\frac{{AC}_{1}-{AC}_{0}}{{AC}_{0}}\times 100$$

where *AC*_1_ and *AC*_0_ are the absorbance of cysHSA and HSA, respectively.

cysHSA molecular mass was evaluated by sodium dodecyl sulfate polyacrylamide gel electrophoresis (SDS–PAGE) on 10% gels. Briefly, HSA and cysHSA samples (2 μg) were prepared in SDS-PAGE loading buffer (65.8 mM Tris-HCl pH 6.8, 2.1% SDS, 26.3% glycerol, and 0.001% Bromophenol blue), boiled for 5 min, and loaded on 10% polyacrylamide gels. Electrophoresis was conducted for 2 h at 120 V, in SDS-PAGE running buffer (25 mM Tris-HCl, 200 mM Glycine, 0.1% SDS, pH 8.3). A protein ladder was used in order to obtain an estimate of the molecular weight of the samples. After electrophoresis, the gel was stained with Coomassie Blue solution (0.5% Coomassie Blue, 10% acetic acid, 40% methanol) and washed with a solution containing 10% acetic acid and 40% methanol before image acquisition using iBright Imaging Systems (ThermoFisher Scientific, Hillsboro, OR, USA).

The isoelectric points of HSA and cysHSA were measured from the pH–zeta potential curves of HSA and cysHSA obtained at different pH values and 25 °C using a dynamic light-scattering instrument (Zetasizer NanoZS, Malvern, UK).

All chemicals were purchased from Merck/Sigma Aldrich, Darmstadt, Germany.

### 3.2. Preparation of FNPs and DOX@FNPs

Ionic nanoparticles, FNPs, were fabricated by the ionic interaction method. In brief, 1.0 mg HA dissolved in 0.5 mL phosphate buffer (PBS, 0.01, pH 7.4) was dropped to 5 mL cysHSA (6.25 mg) in PBS (0.01, pH 7.4).

DLS measurements (Zetasizer NanoZS, Malvern, UK) were performed to estimate the size distribution of FNPs (final concentration 1 mg mL^−1^) at 25 °C. The laser beam operated at 658 nm, while the autocorrelation function was measured at 90°. The polydispersity index (PDI) was obtained from the instrument by fitting the experimental data with the inverse Laplace transformation and Contin methods. Homogenous and mono-disperse populations were claimed for PDI values ≤ 0.3 [52].

Zeta potential values were measured over a 7-day period on a Zetasizer NanoZS, Malvern, UK.

TEM analyses (HRTEM/Tecnai F30 FEI Company, Hillsboro, OR, USA) were performed to determine the morphological properties of nanoparticles. Samples were prepared by placing a drop of nanoparticles on a Cu TEM grid (200 mesh, Plano GmbH, Wetzlar, Germany) and removing the excess sample using a piece of filter paper. Then, a drop of 2% (*w*/*v*) phosphotungstic acid solution was deposited on the carbon grid for 2 min and, once the excess staining agent was removed with filter paper, the samples were air-dried, and the thin film of stained particles was observed with an electron acceleration rate of 80 kV.

All chemicals were purchased from Merck/Sigma-Aldrich, Darmstadt, Germany.

### 3.3. DOX Loading and Release Experiments

DOX-loaded nanoparticles (DOX@FNPs) were prepared by dropping 0.5 mL HA (1.0 mg) in PBS (0.01 M, pH 7.4) to a 5 mL DOX solution (46 μM) in PBS (0.01, pH 7.4) containing cysHSA (6.25 mg). After 1 h at room temperature under magnetic stirring, the suspension was centrifuged (20,000 rpm for 15 min), and the DOX concentration in the supernatant was determined by fluorescence (BioTek Synergy H1 Plate Reader, Agilent Technologies, Santa Clara, CA, USA) using a DOX calibration curve recorded under the same conditions in the 0.8–28 μM concentration range (λ_exc_ = 480 nm; λ_em_ = 590 nm).

The loading efficiency, *LE* (%), was determined according to the following Equation (2):

(2) $$LE\left(\%\right)=\frac{C_{i}-C_{0}}{C_{i}}\times 100$$

with *C_i_* and *C*_0_ being the drug concentration before and after the loading.

A dialysis method under sink conditions was carried out to determine the DOX release profiles from DOX@FNPs. In separate experiments, 2 mL DOX@FNPs dispersion (polymer concentration 1 mg mL^−1^) was loaded in a dialysis bag (SpectraPor, MWCO 3.5 kDa, VWR, Milan, Italy) and dialyzed against 10 mL phosphate buffer (0.01 M, pH 7.4) in either the absence or the presence of 10 mM GSH at 37 °C under constant stirring. At predetermined times, 0.5 mL release medium was withdrawn and replaced with fresh medium. DOX was quantified as reported above.

All chemicals were purchased from Merck/Sigma-Aldrich, Darmstadt, Germany.

### 3.4. Cell Cultures

Breast cell lines MCF7, T-47D, MDA-MB-231, MDA-MB-436, MDA-MB-468, and MCF-10A, as well as hTERT-BJ1 cell lines, were purchased from the American Culture Collection (ATCC, Manassas, VA, USA). MCF7, T-47D, MDA-MB-468, and h-TERT-BJ1 cells were cultured in DMEM High Glucose supplemented with 10% Fetal Bovine Serum (FBS), 2 mM l-glutamine, 1% penicillin/streptomycin, and 1% pyruvate. MDA-MB-231 and MDA-MB-436 cells were cultured in DMEM/F12 supplemented with 10% Fetal Bovine Serum (FBS), 2 mM l-glutamine, and 1% penicillin/streptomycin. MCF-10A cells were cultured in DMEM/F12 supplemented with 5% horse serum (HS), 2 mM l-glutamine, 1% penicillin/streptomycin, 0.5 mg mL^−1^ hydrocortisone, 20 ng mL^−1^ human epidermal growth factor (hEGF), 10 mg/mL insulin, and 0.1 mg mL^−1^ cholera toxin. All cell lines were cultured at 37 °C in 5% CO_2_ in a humidified atmosphere.

All chemicals were purchased from Merck/Sigma-Aldrich, Darmstadt, Germany.

### 3.5. Viability Assay

Cell viability was determined by using the 3-(4,5-dimethyl-2-thiazolyl)-2,5-diphenyl-2H-tetrazolium bromide (MTT) assay and the Sulforhodamine B (SRB) assay as previously described [53,54].

All chemicals were purchased from Merck/Sigma-Aldrich, Darmstadt, Germany.

### 3.6. Metabolic Flux Analysis with the Seahorse XFe96

Real-time extracellular acidification rates (ECARs) and oxygen consumption rates (OCRs) were determined using the Seahorse Extracellular Flux (XFe96) analyzer (Agilent Technologies, Santa Clara, CA, USA). Briefly, 5 × 10^3^ cells per well were seeded into XFe96-well cell culture plates and incubated for 24 h to allow cell attachment. After 24 h, cells were treated with 1μM DOX@FNPs for 72 h. Vehicle-alone (FNPs) control cells were processed in parallel and then washed in pre-warmed XF assay media (or, for OCR measurement, XF assay media supplemented with 10 mM glucose, 1 mM Pyruvate, and 2 mM l-glutamine). Cells were then maintained in 175 μL/well of XF assay media at 37 °C in a non-CO_2_ incubator for 1 h. During the incubation time, we loaded 25 μL of 80 mM glucose, 9 μM oligomycin, and 0.5 M 2-deoxyglucose (for ECAR measurement) or 10 μM oligomycin, 10 μM CCCP, 10 μM rotenone, and 10 μM antimycin A (for OCR measurement) in XF assay media into the injection ports in the XFe96 sensor cartridge. Measurements were normalized by protein content (SRB assay). Data sets were analyzed using XFe96 v2.6 software and Prism 9 software (GraphPad Software, Boston, MA, USA), using two-way ANOVA and the Student’s *t*-test calculations. All experiments were performed in quintuplicate, three times independently.

All chemicals were purchased from Merck/Sigma-Aldrich, Darmstadt, Germany.

### 3.7. Western Blotting

Cells were lysed in RIPA buffer (Merck/Sigma-Aldrich, Darmstadt, Germany) containing one tablet of CompleteTM inhibitor mix (Roche Applied Science, Indianapolis, USA) and one tablet of PhosSTOP™ phosphate inhibitors per 10 mL of buffer and loaded onto SDS-polyacrylamide gels. The gels were transferred to 0.2 µm nitrocellulose membranes, using the Trans-Blot Turbo Transfer System (Bio-Rad, Inc., Milan, Italy). Membranes were incubated with the respective primary antibodies diluted in Tris-buffered saline, 0.1% Tween 20, and 5% bovine serum albumin and incubated overnight at 4 °C. The antigen–antibody complex was detected by incubation of the membranes with IR peroxidase-coupled goat anti-mouse or goat anti-rabbit antibodies and was revealed using the Li-COR system (Li-COR Biosciences, Lincoln, NE, USA). Antibodies and their dilutions used for Western Blot analysis were as follows: mouse anti-human total OXPHOS cocktail 1:1000 (Abcam, Cambridge, UK); mouse anti-β-actin 1:5000 (Abcam, Cambridge, UK).

All chemicals were purchased from Merck/Sigma-Aldrich, Darmstadt, Germany.

### 3.8. Wound-Healing Scratch Assay

TNBC cell lines were seeded into 6-well plates and cultured overnight. Then, cells were treated for 24 h with DOX and DOX@FNPs 0.5 μM, and cell motility was assessed by a wound-healing scratch assay as previously described [55]. Photographs were taken at 10 × magnification using phase-contrast microscopy and are representative of three independent experiments. The wound-healing rate was quantified from the picture using IMAGE J software [56], and standard deviations were determined by Prism 9 software (GraphPad Software, Boston, MA, USA).

### 3.9. Mammosphere Formation Efficiency

A single-cell suspension was prepared using enzymatic and manual disaggregation (25 g needle). Then, cells were plated at a density of 500 cells/cm^2^ in mammosphere medium (DMEM-F12 + 1 × B-27 Plus Supplement + 20 ng mL^−1^ EGF + Pen/Strep) under non-adherent conditions, in culture dishes precoated with 2-hydroxyethylmethacrylate, called “mammosphere plates”. Cells were grown for 5 days and maintained in a humidified incubator at 37 °C. After 5 days of culture, 3D mammospheres > 50 μm were counted using an eyepiece (“graticule”), and the percentage of cells plated with formed spheres was calculated and is referred to as percent mammosphere formation, and it was normalized to one (1 = 100% mammosphere formation efficiency (MFE)). Three-dimensional MFE was analyzed in both the ATP-low and ATP-high subpopulations of cells. All 3D mammosphere experiments were performed in triplicate, at least three times independently.

All chemicals were purchased from Merck/Sigma-Aldrich, Darmstadt, Germany.

### 3.10. qPCR Analysis of CD44 Expression

Two-dimensional cells were grown in 10 cm dishes for 5 days, while three-dimensional cells were grown in 10 cm dishes covered with 2-hydroxyethylmethacrylate for 5 days. Total RNA was isolated using TRIZOL reagent (Invitrogen, Monza, Italy), following the manufacturer’s procedure. Reverse transcription was performed, as previously reported [57], on each RNA sample to generate complementary DNA (cDNA). Gene expression analyses of CD44 (Fw: CAGCAACCCTACTGATGATGACG; Rev: GCCAAGAGGGATGCCAAGATGA) were carried out using the Quant Studio 3 Real-Time PCR System (Life Technologies, Monza, Italy) employing the SsoAdvanced Universal SYBR Green (Bio Rad Laboratories Inc., Milan, Italy), following the manufacturer’s guidelines. Assays were executed in triplicate, and results were normalized using 18S rRNA levels (Fw: AGTCGGAGGTTCGAAGACGAT, Rev: GCGGGTCATGGGAATAACG). The ∆∆Ct method was used to calculate the relative mRNA levels.

All chemicals were purchased from Merck/Sigma-Aldrich, Darmstadt, Germany.

### 3.11. Three-Dimensional Spheroid Formation Assay

Three-dimensional spheroids were generated following the application note by Thermo Fisher (ThermoFisher Scientific, Hillsboro, OR, USA) [58]. Cells were seeded in DMEM/F-12, HEPES, no phenol red, and 1 × B-27 Plus Supplement, 10 ng mL^−1^ of HS bFGF (Gibco Life Technologies, ThermoFisher Scientific, Hillsboro, OR, USA), for a total volume of 0.2 mL/well and a total of 1000 cells/well. The plate was then centrifuged at 120× *g* for 5 min before being placed in a 37 °C, 5% CO_2_ incubator. After 24 h, cells were supplemented with collagen 1 μg mL^−1^, and then the plate was centrifuged again at 120× *g* for 5 min. The spheroids were incubated for 20 days and were observed to form overnight after seeding.

All chemicals were purchased from Merck/Sigma-Aldrich, Darmstadt, Germany.

### 3.12. cBioPortal Analysis

Cell lines’ profiles were extracted from “Cancer Cell Line Encyclopedia from the Broad Institute and Novartis, updated 2019”, using cBioPortal (cbioportal.org, accessed on 1 June 2024). mRNA expression (RNA Seq V2 RSEM) and protein level (protein abundance ratios relative to bridge-sample) of CD44 were evaluated in breast cancer cell lines.

### 3.13. Statistical Analysis

All analyses were performed with Prism 9 (GraphPad Software, Boston, MA, USA). Data were represented as mean ± SD (or ±SEM where indicated). All experiments were conducted at least three times independently, with three or more technical replicates for each experimental condition tested (unless stated otherwise). Statistically significant differences were determined using analysis of variance (ANOVA) tests. For a comparison among multiple groups, two-way ANOVA was used to determine statistical significance. *p* < 0.05 was considered significant.

## 4. Conclusions

In this work, we provided experimental evidence that a new formulation obtained by self-assembly of cysHSA derivative in the presence of HA was able to effectively encapsulate DOX with high efficiency. The structural properties of DOX@FNPs, including size, shape, stability in physiological environments, targeting activity of HA, as well as redox responsivity of cys, were found to make the nanoformulation an ideal candidate for DOX-based breast cancer treatment, increasing the cytotoxic effect of the drug by targeting CD44 protein both in 2D and 3D.

In this regard, it has been found that the carrier was able to modulate the DOX release rate, with the delivered DOX drastically decreasing cellular metabolism by a reduction in both oxygen consumption rate and extracellular acidification rate. Based on this evidence, it has been shown that, compared to free DOX, DOX@FNPs possess a superior ability to reduce cellular viability and motility, as well as spheroid formation.

The use of redox-responsive nanoparticles for the vectorization of DOX to cancer cells is widely explored in the literature, with a huge amount of nanoparticles differing in composition, architecture, and preparation methodologies being proposed. In our approach, we aim to combine the high anticancer efficiency due to the simultaneous redox-responsivity and CD44 targeting ability with the ease of preparation (ionic complexation) allowing for hypothesizing a bench-to-clinic translation with a significant impact on the available DOX-based therapies. Although further experiments are designed for assessing the in vivo efficacy of the nanoformulation, the results presented here could become pioneering in developing therapeutic protocols aiming to reduce chemoresistance phenomena and to increase the site-specificity of DOX in patients with TNBC.

## Data Availability

Data available from the authors upon request.

## Supplementary Materials

The following supporting information can be downloaded at: https://www.mdpi.com/article/10.3390/ijms251910560/s1.
