# Supplementary material for: Novel CD44-Targeted Albumin Nanoparticles: An Innovative Approach to Improve Breast Cancer Treatment
Source: Int J Mol Sci. 2024 Sep 30;25(19):10560. doi: 10.3390/ijms251910560 (PMC11477043; doi:10.3390/ijms251910560)
Supplement: Supplementary file 1 [file ijms-25-10560-s001.zip › ijms-3214078-supplementary.pdf]

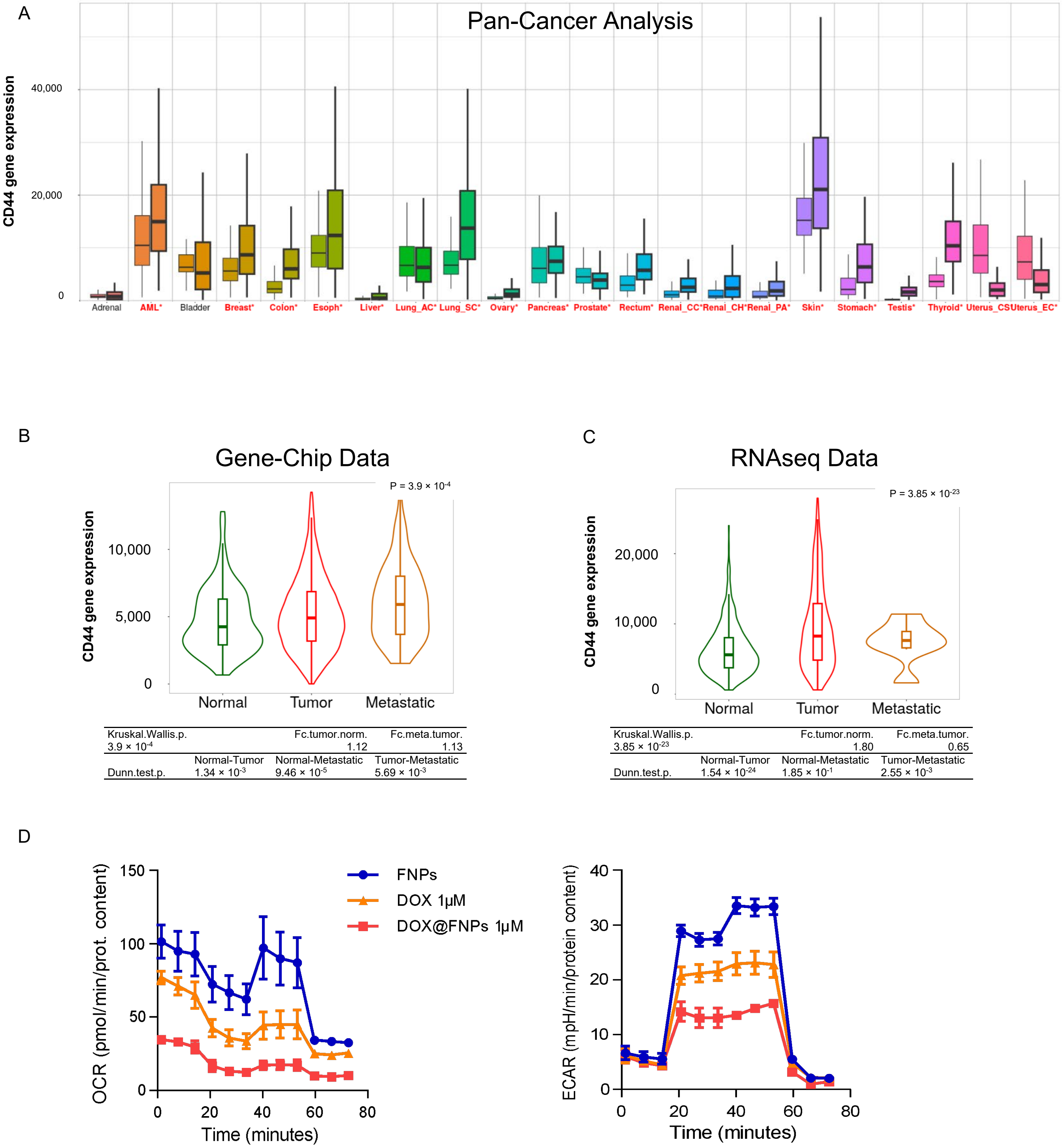

Figure S1. (A) Pan-Cancer analysis of CDK-7 expression between tumor and non-tumor phenotypes (https://tnmplot.com/analysis/). (B) Gene-chip data and (C) RNA-seq of gene and protein expression levels in non-tumor, tumor, and metastatic patients (https://tnmplot.com/analysis/). (D) Metabolic profile of MDA-MB-231 cell line after treatment with DOX and DOX@FNPs. The metabolic profiles obtained with the Seahorse Analyzer XFe96 were represented. OCR and ECAR were monitored in the presence of DOX and DOX@FNPs for 72 h.
